# A Context Analysis with Stakeholders’ Views for Future Implementation of Interventions to Prevent Health Problems Among Employees with a Lower Socioeconomic Position

**DOI:** 10.1007/s10926-021-10010-x

**Published:** 2021-11-03

**Authors:** R. Schaap, F. G. Schaafsma, M. A. Huysmans, A. R. Bosma, C. R. L. Boot, J. R. Anema

**Affiliations:** Department of Public and Occupational Health, Amsterdam Public Health Research Institute, Amsterdam UMC, Vrije Universiteit Amsterdam, Van der Boechorststraat 7, 1081 BT Amsterdam, The Netherlands

**Keywords:** Employees, Lower socioeconomic position, Intervention, Prevention, Implementation science

## Abstract

*Purpose* Health problems among employees with a lower socioeconomic position (SEP) often result from an interplay of problems on multiple life domains. Contextual factors greatly affect implementation of interventions that aim to solve these type of problems. The aim of this study was to gain insight into the organizational and socio-political context for implementation of preventive interventions that consider multiple life domains among employees with a lower SEP. *Methods* In total 16 semi-structured interviews were conducted with stakeholders at organizational level, occupational health service (OHS) level, and at socio-political macro level. Thematic analysis was performed to identify themes that describe the perceptions of stakeholders about the impact of contextual factors on implementation. *Results* The following themes were identified: (1) the importance of addressing problems on multiple life domains among employees with a lower SEP, (2) unclarity of responsibilities for solving problems on multiple life domains, (3) necessity of better collaboration between occupational and curative healthcare, (4) insufficient investments in prevention by employers, (5) difficulties in early identification of employees at risk for health problems, and (6) risk of conflicting role for supervisors in addressing problems on multiple life domains. *Conclusions* Implementation of preventive interventions considering multiple life domains among lower SEP employees is challenging, due to various contextual factors. To improve the feasibility, many different stakeholders both in- and outside occupational health practice need to be involved, collaborate, and need to be convinced of the added value to prevent problems on multiple life domains among employees with a lower SEP.

**Supplementary Information:**

The online version contains supplementary material available at 10.1007/s10926-021-10010-x.

## Introduction

In developed countries there are important health differences between people with a lower and higher socioeconomic position (SEP) [1], which is determined by occupation, education and/or income [2]. People with a lower SEP have a higher risk for health problems, which negatively affects their work participation and increases their risk for premature dropout from the labor market [3, 4]. This points out the importance of preventive interventions that actively support employees with a lower SEP to solve their health problems, who are defined as workers with manual labor (e.g. construction worker) or with lower educated and/or lower income occupations (e.g. administrative worker or truck drivers). In the past decades, many interventions have been developed to prevent health problems among employees with a lower SEP [5–8]. These interventions mainly focused on work and lifestyle related health problems, while health problems among employees with a lower SEP often result from an interplay of problems on multiple life domains, such as unfavorable psychosocial factors and unhealthy living conditions [2, 9, 10].

A complex interplay of problems among employees with a lower SEP, asks for an intervention that can tackle multiple problems in various life domains. For this, the Grip on Health intervention was developed to support employees with a lower SEP to improve their health from a broader perspective, and thereby prevent health problems. This intervention is based on the Participatory Approach (PA) [11], and identifies and solves problems on multiple life domains that affect healthy functioning at work. The current study builds on a pilot study in which the Grip on Health intervention was implemented in occupational health practice and the implementation process was evaluated (not published yet).

The process evaluation focused on factors on the level of the intervention itself (i.e. design and content of the intervention) and the users of the intervention (i.e. employees who received the intervention, and occupational health professionals (OHPs) who facilitated the intervention). The results of the process evaluation showed that the intervention was perceived as relevant by the users of the intervention, but difficult to implement in practice. The next step is to investigate contextual factors (i.e. organizational and socio-political factors) [12, 13]. This can provide more insight into the implementation process of preventive interventions that takes into account multiple life domains among employees with a lower SEP. Research shows that implementation is much more dependent on contextual factors, as opposed to the design and content of interventions [14–16]. Contextual factors are less easy to adjust or influence, and therefore require careful consideration prior to implementation. This means that the implementation of interventions often requires a system approach [17, 18], wherein the complexity of structures and systems in occupational health practice are taken into account. Therefore, this study examined the organizational and socio-political context for implementation of preventive interventions that consider multiple life domains among employees with a lower SEP, and explored contextual factors that affect implementation of these type of interventions.

## Methods

### Study Design

This study used a qualitative, explorative design to obtain in-depth information on the organizational and socio-political context for implementation of preventive health interventions that consider multiple life domains among employees with a lower SEP. The information was obtained by conducting semi-structured interviews among different stakeholders in the organizational and socio-political context of occupational health practice. The Medical Ethics Committee of the VU University Medical Center approved the study protocol and decided that the Medical Research Involving Human Subjects Act does not apply to this study. All stakeholders signed informed consent before participation.

### Context

In the Netherlands, The Working Conditions Act forms the basis for general rights and duties for employers and employees to ensure a safe and healthy working environment. All employers have the obligation to seek support on health and safety from OHPs, that provide professional advice and guidance for a safe and healthy working environment [19]. In case of long term sickness absence of an employee (more than 6 weeks) there is a legal obligation for employers to ask for professional advice from an occupational physician (OP). Also, not sick listed employees have the possibility by law (Working Conditions Act) to ask for advice from an OP without permission from their employer. Furthermore, employers are obligated to offer employees an occupational health examination and evaluate the risks for health and safety at the workplace. OHPs can either be self-employed or employed by occupational health services (OHSs). OHSs or self-employed OHPs offer various types of contracts to employers, such as rather basic contracts in which only advice is provided on a single occasion at the request of the employer, up to contracts with continuous in-house services of multiple OHPs. Moreover, employers can also have an in-house OHS. The Works Council or employees representatives must approve the content of contracts with the OHS. In practice, the content of these contracts vary widely, however there are still employers that do not fulfill the Working Conditions Act or do not have any contract at all [20, 21].

**Fig. 1:**
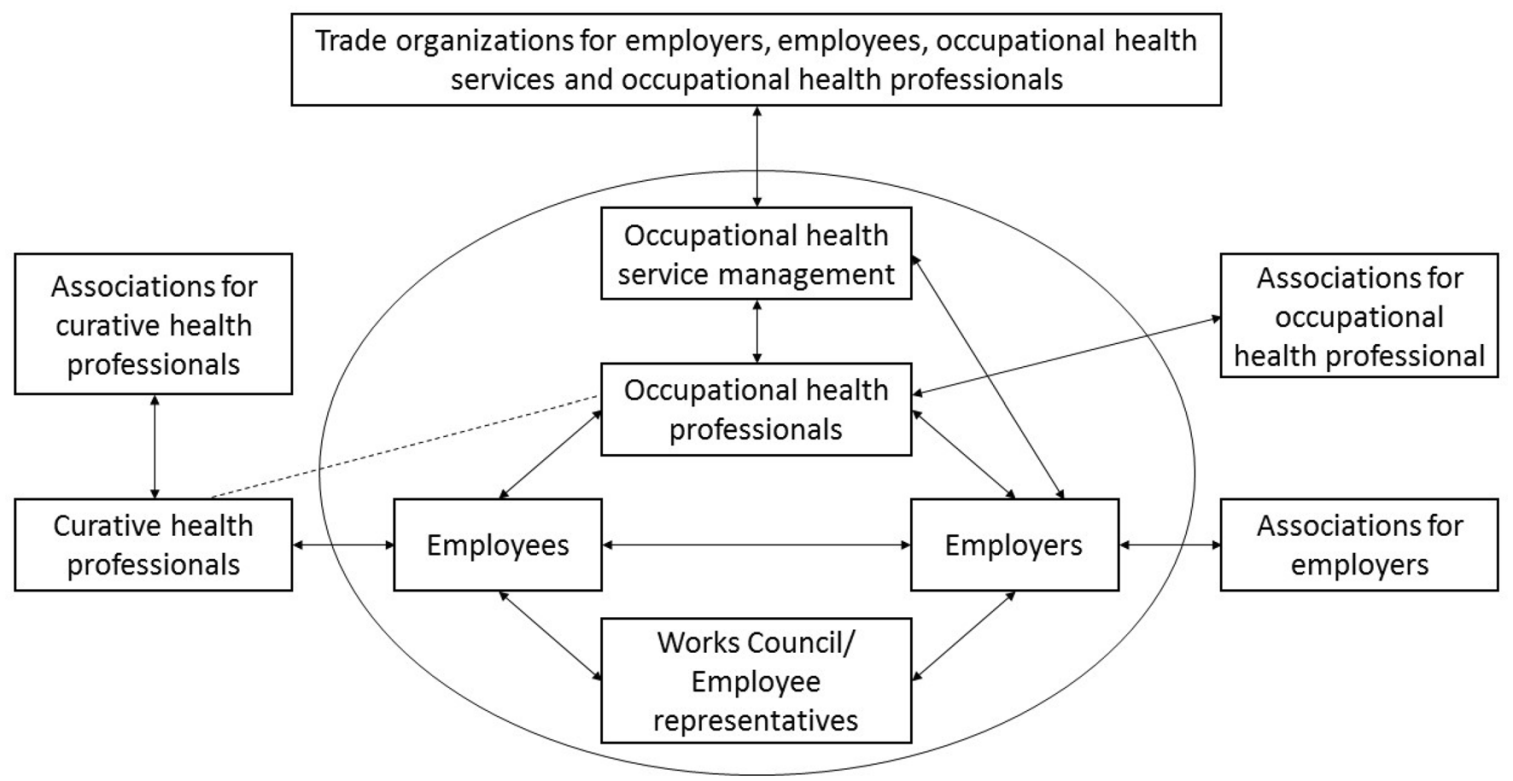
Relevant stakeholders in (occupational) health practice in the Netherlands

In summary, employers and employees are both responsible for healthy and safe working conditions in an organization. Sometimes employees in an organization are represented by a Works Council or employee representative. Employers and employees receive advice from OHPs and OHS managers on how to achieve a healthy and safe working environment. OHPs and employers are represented by OHPs associations and employer associations. There are also trade organizations that inform and support employees, employers, OHPs and/or OHSs. Evidently, employees can also visit a health professional in curative healthcare (e.g. general practitioner (GP)), and these professionals are also represented by associations. Relevant stakeholders in (occupational) health practice in the Netherlands are shown in Fig. 1.

### Recruitment

Stakeholders were selected within the organizational and socio-political context of occupational health practice in the Netherlands and were divided in three type of levels; (1) organizational level, (2) OHS level and (3) socio-political macro level. The organizational level contains stakeholders that work for an organization or company with lower SEP employees and focus on improving and maintaining the health and safety of employees in an organization (e.g. human resource manager or manager health and safety). The OHS level contains stakeholders that work for an OHS (e.g. manger OHS) and focus on supporting organizations in achieving a healthy and safe working environment. The socio-political macro level contains stakeholders that work for an organization that provides support or advice on healthy and safe working conditions at a higher level than stakeholders working for an OHS (e.g. representative of trade association). Stakeholders at organizational level are part of the organizational context, and stakeholders at OHS and socio-political macro level are both part of the socio-political context of occupational health practice. To recruit stakeholders on different levels, we used a combination of purposive and snowball sampling. For purposive sampling, stakeholders needed to have a profession related to occupational health (e.g. manager health and safety) and they must represent a relevant stakeholder in occupational health practice in the Netherlands, as shown in Fig. 1. Stakeholders meeting the predefined criteria were approached by using existing contacts of the research team—i.e. snowball sampling. Stakeholders were invited by email and provided with a short description of the aim of the study. If stakeholders had additional questions about the study, the primary researcher (RS) answered these questions during the interview. In total, 16 semi-structured interviews were conducted; three with stakeholders at organizational level, four with stakeholders at OHS level and nine with stakeholders at socio-political macro level (see Table 1).

**Table 1:** Stakeholders

Levels	Stakeholders
Organizational level	HR manager logistic company
	HR advisor facility department hospital
	Manager health and vitality steel company
Occupational health service level	Department coordinator occupational social workers
	Department coordinator occupational nurses
	Two managers of an occupational health service
Socio-political macro level	Policy officer Netherlands Trade Union Confederation
	Board member Royal Dutch Medical Association
	Representative guideline development & research Dutch College of General Practitioners
	Board member Dutch Association of occupational labor experts
	Two policy officers Confederation of Netherlands Industry and Employers
	Board member Netherlands Society of Occupational Medicine
	Board member trade association for service providers of occupational health care
	Three policy officers trade association for organizations in the construction sector
	Representative trade association for service providers of occupational health care

### Data Collection

Semi-structured interviews were conducted by telephone or video-conference between May and November 2020. A topic guide was used to examine the perceptions of stakeholders on preventive health interventions that consider multiple life domains and to explore related contextual factors. The following topics were discussed: (1) addressing problems on multiple life domains in occupational health practice (2) how problems on multiple life domains are dealt with and which stakeholders play a role in dealing with these problems; (3) the extent to which prevention is important in occupational health practice, (4) the implementation of preventive services in organizations; (5) collaboration between organizations, OHSs and OHPs in occupational health practice; (6) the organization of occupational healthcare in the Netherlands in relation to addressing problems on multiple life domains; and (7) the collaboration between occupational healthcare and curative healthcare. Within these topics, questions were based on contextual factors that could affect implementation, which were identified by Fleuren et al. [12]. Furthermore, in case employees with a lower SEP were discussed within these topics, this group of employees were conceptualized as workers with blue-collar occupations or a lower educational level, who more often have health problems on multiple life domains and an increased risk to drop out of the labor market, as compared to workers with white-collar occupations or a higher educational level. For each stakeholder the interview topics were the same, but questions were stakeholder-specific to align the questions to the profession and background of the stakeholder. Interviews lasted around 30–60 min and were conducted in Dutch by the primary researcher (RS).

### Data Analysis

The interviews were audiotaped and transcribed verbatim. The data was coded using Atlast.ti. Thematic analysis was used to analyse the data [22]. The analysis started with re-reading the transcripts, listening to audio-tapes and making summaries of each transcript to become familiar with the data. Thereafter, open coding of the transcripts was performed using an inductive approach. During this process an initial list of codes was produced by the first coder (RS). Another coder (FS) read several transcripts and checked the codes. Next, the data was searched for similarities and discrepancies to combine and group codes. There were several meetings to discuss and categorize the codes into sub-themes (RS, FS, MH). This ultimately resulted into broader themes, which were depicted in code matrices. After 12 interviews, the themes were discussed with the whole research team (RS, FS, MH, CB, JA), wherein we came to the conclusion that we did not yet achieved data saturation. Some underlying factors influencing implementation were still unclear and it became clear that trade associations were an important stakeholder that were not yet interviewed. Therefore, four extra interviews, of which three with representatives of trade associations, were additionally performed to achieve data-saturation. Open coding was performed for the additionally performed interviews. Another coder (AB) also read several transcripts and checked the codes. The remaining steps were repeated to adjust and finalize codes, (sub-)themes and code matrices. Moreover, sub-themes were categorized according to stakeholder level, which provided an overview of similarities and discrepancies between stakeholder levels for the different sub-themes. The last stage consisted of meetings with the whole research team to reach consensus on the final themes.

## Results

Themes were identified on the perceptions of stakeholders on the impact of the organizational and socio-political context for implementation of preventive health interventions that consider multiple life domains among employees with a lower SEP. These themes also include contextual factors that may facilitate or impede implementation in occupational health practice. The different themes and related contextual factors are presented in Table 2 and discussed below. An overview of themes, sub-themes and codes can be found in Table 1 of the supplementary materials, wherein the stakeholder level that endorsed a sub-theme was also described.

**Table 2:** Overview of themes and related contextual factors

Theme	Contextual factors
The importance of addressing problems on multiple life domains among employees with a lower SEP	• Problems on multiple life domains are recognized among employees with a lower SEP • Addressing problems among employees with a lower SEP requires more attention • Difficulty to solve problems on multiple life domains among employees with a lower SEP • Employees with a lower SEP are hard to reach for participation in preventive interventions
Unclarity of responsibilities for solving problems on multiple life domains	• Low sense of responsibility experienced in occupational health services • Employers eventually determine the content of occupational services provided • Employers who see their employees as valuable feel responsible • Employers with sufficient resources feel responsible • Employers of employees with a lower SEP do not always act in the interests of employees • Low sense of responsibility experienced in small and medium sized enterprises • Limited influence of employees with a lower SEP on occupational health policies • Low sense of responsibility experienced in occupational and curative healthcare
Necessity of better collaboration between occupational and curative health care	• Two separate options to discuss problems on multiple life domains • Lack of collaboration between occupational and curative healthcare • Collaboration between occupational and curative healthcare is perceived difficult
Insufficient investments in prevention by employers	• Prevention of health problems and (long-term) sick leave is an important priority • Lack of attention for prevention in contracts • Less resources for prevention in smaller organizations or organizations in a crisis • Employers not seeing their employees as valuable invest less in prevention • Results of prevention are often unclear and cannot always be quantified • Employers focus on short term results and only act in case there are problems • Employers without support from key stakeholders in organizations difficult to convince to invest in prevention
Difficulties in early identification of employees at risk for health problems	• Methods for the identification of employees at risk mainly focus on indicated prevention • Limited availability of occupational physicians to preventively address problems on multiple life domains • Occupational social workers or occupational nurses more accessible than occupational physicians to preventively address problems on multiple life domains • Organizations not always willing to invest in preventive conversations with occupational health professionals or in preventive interventions
Risk of conflicting role for supervisors in addressing problems on multiple life domains	• Supervisors play an important role in the early identification of workers at risk for health problems • Supervisors play an important role in referring employees to an OHP on time • Supervisors discussing problems on multiple life domains may disadvantage employees • Privacy regulations to discuss problems on multiple life domains are unclear

### The Importance of Addressing Problems on Multiple Life Domains Among Employees with a Lower SEP

The majority of the stakeholders recognized that employees with a lower SEP more often have problems on multiple life domains, as opposed to employees with a higher SEP. Although, several stakeholders representing all three levels described that employees with a higher SEP also encounter problems on multiple life domains, it was more important to address these problems among employees with a lower SEP. Employees with a lower SEP more often have unhealthy working and living conditions, and other problems in- and outside the workplace, such as unhealthy lifestyles or financial problems. Problems pile up and could further accumulate if not addressed on time, which makes it even harder to solve problems. As a result, stakeholders at all three levels described that employees with a lower SEP risk ending up in a negative spiral, wherein one problem perpetuates another problem or one problem makes it difficult to solve another problem.

*S9 (socio-political macro level): “Yes, it more often leads to problems, in particular because it is not one aspect, it is often a accumulation of, and then lifestyle has a more negative effect. And there are more things that make them vulnerable, and these things are also interrelated. So lifestyle can be hard, because they may need an investment or money to solve that, and if you have a low income or struggling to make ends meet, than you will not work on that (lifestyle), while your health is getting worse, and with a worse health they may find it difficult to get a job, you can see that the vicious cycle arises”.*

The majority of the stakeholders expressed that employees with a lower SEP need more support in case they have problems on multiple life domains. Some stakeholders representing all three levels mentioned that this group of employees experience difficulties with finding the right health professional to support them in solving their problems, as there are many different professionals working at different health organizations. Some stakeholders at socio-political macro level stated that it is more difficult for them to get an overview of their problems on multiple life domains. Another stakeholder of an OHS described that they need more support, as they are less surrounded with people in their environment in- and outside the workplace that can help to solve their problems.

*S5 (OHS): “Often they do not see a solution and they are in their own bubble, but that occurs to everyone, the moment that you are completely in your own bubble, then you cannot look beyond that bubble, and yes, the moment that you are regularly stimulated by your collegaues and your relatives to achieve behavioral change, well then you start thinking about that. In this group you often see that such stimulus does not come from the environment, because everyone is in the same type of bubble.”*

Several stakeholders representing all three levels mentioned that employees with a lower SEP are difficult to reach for participation in preventive health interventions. Stakeholders at organizational and socio-political macro level mentioned that employees with a lower SEP do not easily ask for help and do not like to talk openly about their problems, due to for example mistrust in the workplace, or a certain group dynamic or culture at the workplace to keep on going, and not to complain. Though, some stakeholders at socio-political macro level stated that employees in general don’t see the added value to participate in preventive health interventions when they do not experience any health complaints.

### Unclarity of Responsibilities for Solving Problems on Multiple Life Domains

All stakeholders of an OHS expressed not being responsible to solve problems on other life domains than work. OHSs stated that these type of problems are discussed by OHPs, but no actions are taken to actually solve these problems. Many stakeholders, including OHSs themselves, indicated that OHSs are commercial organizatons that sell services to employers related to work and health issues, and that the content of OHSs services are eventually determined by the employer. Some stakeholders representing all three levels mentioned that services from OHSs are mainly perceived as an advice and that OHPs are seen as advisors for employers:

*S7 (OHS): “But we have a responsibility to give the right advice to both the employer and the employee. So, we have, it might be good for you to realize that, we have obviously as an occupational health service, we do not have care tasks like a hospital. It is actually, an occupational health service is not a healthcare facility and we deliver business-to-business services. We deliver services to an employer that happen to be care related, and as an occupational health physician you have a legal obligation to deliver care, but in fact, it is mainly an advice what you deliver.”*

Several stakeholders, including stakeholders at organizational level, mentioned that some employers feel a responsibility to solve problems outside the workplace. Some of these stakeholders stated that these type of employers see their employees as valuable. Feelings of responsibility by employers, increases the opportunity to deal with problems outside the workplace (e.g. sleep workshops) and facilitate solutions that are provided by external services or interventions (e.g. support for financial problems). A few stakeholders stated that mainly large organizations with sufficient resources facilitate solutions, which are offered in the form of a menu (e.g. lifestyle interventions, support from a psychologist or social worker) where employees can choose from. Smaller organizations may experience difficulties with the funding of solutions for problems outside the workplace:

*S4 (OHS): “but my first reaction would be just a lack of resources, or at least the choice to use these resources for this. I think it is easier for large companies, that financing is simply easier”*.

Several stakeholders at OHS and socio-political macro level stated that there are also employers not feeling responsible to solve problems outside the workplace. Some of these stakeholders mentioned that this is especially true for employers of employees with a lower SEP, who do not see their employees as valuable and are putting economic interests first. Some of these stakeholders also mentioned that some employers quickly point to problems outside the workplace as a main cause for sick leave. Stakeholders representing small and medium sized enterprises (SMEs) stated that employers of SMEs in general do not feel responsible to solve problems on multiple life domains, but that it is the responsibility of the OHS or employees themselves. A SME employer does not have much expertise on health related problems, and therefore completely relies on the services of an OHS:

*S12 (socio-political macro level): “In general you must say that the willingness to pay for that themselves is very low, because the entrepreneur thinks it is not their responsibility, but the responsibility of the external, and last but not least from the employee himself.”*

Some other stakeholders representing an organization and GPs also stated that eventually employees are responsible to solve their own problems, and that employers or OHPs can only offer tools. Some stakeholders at socio-political macro level mentioned that the extent to which an employer acts in the interest of their employees is dependent on the influence of employees on occupational health policies in organizations. One stakeholder representing OPs in the Netherlands stated that in organizations with a vast majority of lower SEP employees, employees have a limited influence and are often poorly represented. Consequently, these type of employers have less attention to solve problems on other domains than work:

*S13 (socio-political macro level): “What I see is that the higher educated people are, the more empowered the employees are, the more actively they play a role in organizational policies, so influencing how it happens, the better these type of questions are considered. So yeah, people with a lower SEP, often lower educated, I have collected some examples over the course of 30 years that I am an occupational physician, and it were always the lower educated, often people with an immigrant background, sometimes with a small language problem, lower skilled positions, those were often treated the worst.”*

Several stakeholders at socio-political macro level expressed that neither occupational nor curative healthcare feels responsible to solve problems on multiple life domains. A few stakeholders at socio-political macro level stated that OPs must focus on solving work-related problems, and one stakeholder representing GPs stated that GPs must focus on solving health complaints. Several stakeholders described that GPs have limited expertise and time to discuss work-related problems. Therefore, one stakeholder representing GPs suggested that general practice nurses have more time and may be more suited to solve these problems in curative healthcare. Last, all representatives from trade associations expressed not feeling responsible to solve problems on multiple life domains, they only give advice or share knowledge with employers, OHSs and OHPs.

### Necessity of Better Collaboration Between Occupational and Curative Healthcare

There are two options to discuss problems on multiple life domains, either through occupational healthcare or curative healthcare. Some stakeholders representing GPs and OPs in the Netherlands struggled with the fact that occupational healthcare is separated from curative care. OPs are paid by employers and feel that they are positioned outside the curative healthcare system, making the collaboration between occupational and curative healthcare difficult, as was experienced by many more stakeholders:

*S9 (socio-political macro level): “But we see the problem that occupational medicine, and also insurance medicine, that they are now completely separate, so in terms of financing and other such, this will cause problems in terms of collaboration, collaboration with a general practitioner or with anyone else. So, given the implementation of care, being able to collaborate, it is an obstacle how it is currently organized. And this is what we mean. So, we actually aim for de-seperation and to work towards integrated care.”*

The majority of the stakeholders pointed out that problems on multiple life domains can usually be discussed at the workplace. For this, an open and safe culture within the organisation is essential, as was stated by some stakeholders representing trade associations. Other stakeholders at OHS and socio-political macro level also mentioned that problems on multiple life domains can or should be discussed in curative healthcare. To actually solve these problems many stakeholders stressed the importance for a better collaboration between occupational and curative healthcare. Collaboration is needed because several stakeholders at OHS and socio-political macro level indicated that GPs are often the first or only health professionals to contact in case of health complaints, especially for employees who are self-employed and cannot contact an OHP through their employer. But, GPs do not always consider the relation between health complaints and work and do not always know how to collaborate with OPs:

*S8 (socio-political macro level): “The collaboration between the general practitioner and occupational physician really needs to be improved and employees often go, also completely justified, first to their general practitioner when they have health complaints, and a general practitioner is often, how do you say that, unable to recognize what’s going on considering their job. So, the collaboration between the occupational physician and general practioner must be improved and the collaboration, if it happens, will also be of benefit for the employee.”*

The possibilities for improving the collaboration between occupational and curative healthcare that were suggested by many stakeholders focus on integrated care. Some stakeholders mentioned that we should organize healthcare around an individual employee (network care), others mentioned that we should integrate an OP in curative healthcare, or that work factors should be taken into account in curative healthcare.

### Insufficient Investments in Prevention by Employers

The majority of the stakeholders acknowledged that prevention of health problems and (long-term) sick leave is an important priority. However, some stakeholders also mentioned that much more attention is needed for prevention than currently is the case, also in the education of health professionals. Several stakeholders, including trade organizations themselves, mentioned that trade organizations can play an important role in increasing the attention for prevention in organizations. Preventive services that are offered in an organization depend on the contracts between an employer and OHS. Several stakeholders representing all three levels described that preventive services are often not included in the basic contracts, and that basic contracts mainly focus on the guidance of employees on long term sick leave:

*S8 (socio-political macro level): “The occupational health service or the occupational physician, they have a contract with the employer, only within that contract there is actually very little arranged in the field of prevention, unfortunately it is mainly about the guidance of employees on sick leave, while we would like to see that prevention is also part of that contract, only that happens far too little and we think that’s a shame.”*

In addition, some stakeholders representing trade organizations stated that there is less attention for prevention in contracts due to the Gatekeepers Act. This law shifted the attention from prevention to the guidance of employees on sick leave. Another reason, mentioned by some stakeholders representing OPs, is that OHPs are not always involved in the formation contracts between an OHS and employer. OHPs that are more involved in this process are more likely to be used for preventive services in organizations.

Stakeholders described several reasons for employers to spend money on prevention. According to the majority of the stakeholders, financial resources play a major role in the decision to implement preventive services. Smaller organizations or organizations in an economic crisis (e.g. due to the Covid-19 pandemic) have less resources (time and money) to invest in preventive services. As a result, employers first invest in services that focus on the guidance of employees on long term sick leave. Second, stakeholders mentioned that employers who do not see their employees as valuable, also tend to invest less in prevention. Third, the extent to which the results of prevention are visible and provide a return on investment is also important for the majority of the stakeholders. But, the results of prevention are often unclear and these results cannot always be quantified, making it hard to convince employers to invest in prevention. Fourth, some stakeholders at OHS and socio-political macro level stated that employers focus on short term results as they are not or less aware of the benefits of prevention on the longer term. Several stakeholders at socio-political macro level do not agree with that, as they mentioned that is not a matter of not knowing the benefits, but a matter of employers not wanting to invest in prevention:

*S13 (socio-political macro level): “It is a kind of primarily human behavior that we struggle to distinguish long term goals from short term investments. You see it everywhere, even in the whole establishment of prevention. We have a ministry of health, but nearly 100 billion is going to curative healthcare and very little is going to preventive care. I always say, you can also see it in society, if your house is on fire then the fire fighters come, and we all pay for it, that is publicly funded, so curative. But if the same fire fighter rings the doorbell the night before the big fire and says: can I give you some advice about escape routes and other things, then you have to pay for it yourself. It is very complicated and apparently we have the tendency to see the dangers and then pay for it.”*

Several stakeholders at socio-political macro level stated that we need to work out business cases and develop innovative preventive services to convince employers to invest in prevention. At last, several stakeholders explained that the amount of support from key stakeholders in organizations for prevention (e.g. supervisor, HR manager) is just as important. If there is no support from key stakeholders for prevention, it was mentioned that it is very hard to convince employers to invest in prevention.

### Difficulties in Early Identification of Employees at Risk for Health Problems

The majority of the stakeholders mentioned that methods for the identification of employees at risk mainly focus on indicated prevention (i.e. target high risk employees to prevent health problems). Therefore, employees are mainly identified when they may already experience health complaints and are at risk for health problems. This makes it very difficult to identify employees before they have problems on multiple life domains. Several stakeholders at organizational and socio-political macro level indicated that we should address problems on multiple life domains preventively by having conversations with employees regularly:

*S14 (socio-political macro level): “Just have regular conversations with these people about how their lives work, to tackle or even prevent problems as quickly as possible. But prevention is always difficult. So at least tackle it as quickly as possible, and in the context of sustainable employability to prevent them from falling through the ice.”*

Some stakeholders representing all three levels described that the availability of OPs in practice is limited and there is usually not enough time to solve problems on multiple life domains. Some stakeholders of OHSs mentioned that occupational social workers or occupational nurses usually have more time and are more accessible to discuss problems on multiple life domains preventively. Many stakeholders stated that organizations that performed an preventive occupational health examination, also offered individual follow-up conversations or preventive interventions. However, several stakeholders at OHS and socio-political macro level also noted that organizations often do not perform these types of follow-ups, as they are not always willing to invest money in follow-ups.

### Risk of Conflicting Role for Supervisors in Addressing Problems on Multiple Life Domains

Several stakeholders representing all three levels stated that supervisors play an important role in the early identification of employees at risk for health problems. This way, supervisors can refer employees to an OHP on time or take other necessary actions to prevent sick leave. Some stakeholders, including the stakeholders at organizational level, mentioned that supervisors not only play a role in the identification of problems, but also have regular conversations with employees. Based on these conversations supervisors can determine whether an employee needs support of an OHP in solving problems:

*S1 (organization): “Not to say: how is it going at your work, you are doing well or not, but how are you really doing? And then from that perspective, stick the feelers’ out to see whether, okay is he still feeling well, if not, what is the reason, as far as the employee wants to share that, and then offer a helping hand, if there is actually help needed, in whatever form, then we do have an occupational health service available.”*

One stakeholder of an organization mentioned that a positive consequence of supervisors having regular conversations with employees, is that they are more likely to talk with their supervisors about problems. Not every supervisor is able to perform this type of conversation, and therefore several stakeholders from organizations and OHSs mentioned that there is a lot of attention for training of supervisors in early identification of problems and performing preventive conversations with employees. Some stakeholders representing all three levels also described that giving supervisors a more prominent role in the guidance of employees (i.e. self-management model), improves supervisors’ responsibility for employees’ health and safety at the workplace. Other stakeholders mentioned that supervisors taking responsibility may also unintentionally disadvantage employees; supervisors may take on the role of an OHP which may not always be the desired situation, support from an OHP may come too late, and supervisors may take advantage of privacy-sensitive information of employees due to the unequal relationship between an supervisor and employee:

*S13 (socio-political macro level): “And I think that is quite a disturbing development, because then you have to remember that this happens constantly in an unequal relationship. The employment relationship is simply one where the employer has obvious authority, so there is an unequal relationship where an employee often acts submissive to what an employer expects, and certainly people with a low socioeconomic status. Because your contract could not be extended, or you will receive a bad evaluation. And that also results in a rather complicated and therefore not properply regulated domain for which I have no solution, but I do see the risks.”*

Therefore, several stakeholders described that an OHP is important to advise and guide employees, independently of other interests. Moreover, some stakeholders stated that it is difficult for supervisors to be fully responsible for employees’ health and safety, as they are not allowed to discuss health related problems with employees. Though, some stakeholders at organizational and socio-political macro level described that employees usually discuss everything with their supervisor, and that the privacy regulations with regard to discussing problems on multiple life domains are unclear.

## Discussion

This study described the perceptions of different stakeholders on the context for implementation of preventive interventions that consider multiple life domains among employees with a lower SEP. Many organizational and socio-political factors were identified which impede or facilitate implementation and are related to the following themes; (1) the importance of addressing problems on multiple life domains among employees with a lower SEP, (2) unclarity of responsibilities for solving problems on multiple life domains, (3) necessity of better collaboration between occupational and curative healthcare, (4) insufficient investments in prevention by employers, (5) difficulties in early identification of employees at risk for health problems, and (6) risk of conflicting role for supervisors in addressing problems on multiple life domains.

This study showed that problems on multiple life domains are considered important to address among employees with a lower SEP. However, stakeholders in this study described that this group of employees has more difficult circumstances in- and outside the workplace to solve their problems. Stakeholders also mentioned that employees with a lower SEP more often have an accumulation of problems that are interrelated and maintain one another. As was described in this study and in literature, they could end up in a vicious cycle, which makes it even more difficult to solve problems [23]. Hence, this group of employees needs support to break this cycle, but it remains uncertain whether all problems on multiple life domains can be addressed at the workplace. Findings of this study showed that responsibilities of all stakeholders involved to solve problems on multiple life domains are considered unclear. In the Netherlands, occupational healthcare is operating by law in a private market and strongly depends on the contract between an OHS and employer. OHSs are often commercial organizations and they do not feel the responsibility to solve problems on other life domains than work. In the end, employers determine the intensity and focus of services provided by the OHS, which may be a barrier for preventive interventions that consider multiple life domains. Although there are some legal obligations for employers, stakeholders in this study described that employers mainly focus on the guidance of employees on sick leave and to a lesser extent on the prevention of sicke leave. Moreover, it is evident that not all employers feel responsible and are willing to pay for solving all kinds of problems preventively. This may, to some extent, also apply to other countries, because international studies show that the in the majority of the countries OHSs are paid mainly or only by employers [24, 25].

Stakeholders in this study representing GPs and OPs also mentioned that they do not feel responsible to solve problems on multiple life domains. In the Netherlands, occupational and curative healthcare are strictly separated. This provides employees two options to discuss their health complaints, but a connection between occupational and curative healthcare to collaborate is missing. The financial systems of occupational and curative healthcare are also separated in the Netherlands, which may further discourage collaboration. In contrast, occupational and curative healthcare are not strictly separated in other European countries, such as Finland and Germany [26]. In these countries, OPs and GPs are often the same person or both OPs and GPs can perform occupational and curative tasks. For example, in Finland occupational health services are important providers of curative healthcare. Finnish OPs partly act as GPs for employees, about half of the GP visits takes place within occupational healthcare and almost all visits to an OP were for primary care advice [27]. Hence, to provide adequate care to employees, European countries, such as Finland and Germany, are less dependent on collaboration between occupational and curative healthcare. Unfortunately, in the Netherlands adequate care for employees is highly dependent on collaboration between occupational and curative healthcare. Collaboration between GPs and OPs in general is not optimal [28, 29], and this is also a problem in countries where GPs are certified to give advice on sick leave [30–32]. Although, GPs are often the first health professional for employees to discuss health complaints, they are reluctant to discuss work-related problems, due to a lack of expertise and time [26, 28, 33]. Moreover, GPs express reluctance to contact an OP due to a lack of confidence in the independence of OPs and limited access of OPs [28, 32]. Thus, collaboration between these two domains needs improvement. To improve the collaboration, the first step is to raise awareness among GPs on the relation between health complaints and work, to train GPs to be more able to discuss work-related problems, and to refer patients more easily to an OP [28, 31, 32, 34]. The second step is to explore initiatives to improve the collaboration, by for example addressing misconceptions between GPs and OPs roles and independence of OPs and how to reach and communicate effectively with each other [31].

In this study, several stakeholders described that employers of employees with a lower SEP give the health of their employees less priority and often put economic interests first. They focus more on the organizational processes and performances of employees than on the health and well-being of their employees [35–37]. Although, it is understandable that employers primarily think about the needs and interests of their businesses, most employers are still willing to ensure a safe and healthy working environment for their employees. However, literature shows that some employers are more reluctant to invest in the working environment, particularly in case the employer considers employees with a lower SEP to be of lower value and more easily replaceable [38]. Prevention is considered an important priority by all stakeholders in this study, but they also mentioned that investments in prevention are limited. Literature shows that there is insufficient attention by employers for prevention [29, 39], that a low number of organizations has policies on prevention, and if there are policies on prevention these are mainly present in larger organizations [39]. The latter was also found in the present study; a smaller organization with fewer resources can be considered a barrier for investments in prevention. Another explanation for insufficient investments described in this study and in literature, is that the benefits of preventing health problems on the longer term are unclear [29]. In the Netherlands, employers pay and therefore determine which preventive services and services for sick-listed employees are provided to employees in organizations. However, sick listed employees result in a financial burden for employers and the implementaton of services for these employees are linked to short term economic benefits [38]. As a result, employers are inclined to mainly focus on services for employees on sick leave [29], and will less likely invest in preventive services.

Prevention in organizations is challenging, as this study showed that methods for the identification of employees mainly focus on indicated prevention, which makes it difficult to early identify employees at risk. Although, employees in the Netherlands are enabled by law to visit an OP for preventive advice, employees make little use of this opportunity. Moreover, OPs availability to preventively solve problems on multiple life domains was also considered limited in this study. Their tasks mainly consist of providing advice to employees on sick leave, in which employees may be unfamiliar with the preventive role of OPs [40]. GPs also have limited time and expertise [32], and therefore the option for OPs to collaborate with other health professionals that are more accesible to solve problems on multiple life domains should be further explored. Literature also shows that employees may have a negative attitude towards OPs [40, 41]. They are still insufficiently convinced of the OPs independence, and see them as someone that is on the side of the employer as they are contracted and paid by employers [29, 40, 41]. In contrast, employees in Finland are very satisfied with the services of an OP and visiting an OP is more common than visiting a GP, partially due to good accessibility of OPs [42, 43]. Possible options that may change the attitude of employees towards OPs, improve the accessibility of OPs and the collaboration with other health professionals are: (1) integrate an OP or other professional specialised in work-related health problems in curative healthcare, or (2) organize healthcare around an individual person [29, 31, 39].

Supervisors in this study were also considered important for the early identification of employees at risk. Supervisors have regular contact (sometimes daily) with their employees, and could therefore be the first person to notice whether an employee is at risk and refer them to an OHP at an early stage. Multiple studies showed that supervisor support is an important resource for health and well-being at work [44, 45]. Supervisors that support employees to overcome health-related problems could violate the privacy regulations [46], but according to some stakeholders in this study this legal barrier was not seen as a barrier in practice, showing that the privacy regulations with regard to problems on multiple life domains are unclear. Other stakeholders in this study described that this may also result in unwanted situations for employees, because of the hierarchical relationship between an employee and supervisor. Whether supervisors can discuss health-related problems with their employees strongly depends on the organizational culture, and the relation between supervisors and employees [47].

### Strengths and Limitations

This qualitative study provided in-depth information about organizational and socio-political factors in occupational health practice among different stakeholders. Different contextual factors were identified, which provide valuable information for future implementation of preventive interventions that consider multiple life domains among employees with a lower SEP. Furthermore, this study seems context specific, but factors found in this study were also found in studies conducted in other countries, thus suggesting transferability of findings. A limitation of this study is that stakeholders were partially recruited by the use of snowball sampling, which could result in a sample of stakeholders that were already interested in the topic of this study and may hold more positive views on their own role in implementation. Another limitation related to the sample of stakeholders is that contextual factors in relation to the prevention of health problems among employees with a lower SEP were discussed with stakeholders on organizational and socio-political level. The perspective of employees with a lower SEP is missing, while literature shows that stakeholders may hold other, or even more negative views on employees, than employees themselves [48, 49]. A last limitation is that factors related to the content of interventions and potential users of interventions (e.g. occupational health professionals) were not investigated, but may in practice interfere with organizational and socio-political factors. For example, the degree to which the user is able to use the intervention in daily practice, may influence the degree to which organizations are willing to support implementation.

### Implications for Research and Practice

Due to the difficulty to solve problems on multiple life domains among employees with a lower SEP, further research is needed on how organizations can adequately reach and support lower SEP employees with problems on multiple life domains. Furthermore, in this study employees with a lower SEP consisted of people with a regular job. However, employees with an even lower SEP, such as precarious workers or without a job, possibly have more problems on multiple life domains. Therefore, further research is also needed on the perspectives of stakeholders on employees with an even lower SEP. In addition, it should be explored which stakeholder could best deliver preventive interventions that consider problems on multiple life domains. Currently, the responsibilities are unclear, forming a situation wherein nobody feels responsible for dealing with problems on multiple life domains. Many different stakeholders, both in occupational and curative healthcare, are involved in dealing with these problems, but to effectively address problems on multiple life domains improvement in collaboration between these stakeholders is needed. To achieve this, reorganization of the Dutch healthcare system may be required towards more integrated care [29], wherein an employee is not dependent on the services of an employer and focus is on functioning of an individual in all life domains. Integrated care also has implications for the financial systems of both occupational and curative healthcare. Thus, to further improve collaboration the government needs to explore on how to financially bring these systems together or to financially compensate collaboration. Although, these separated healthcare systems make it difficult to effectively address problems on multiple life domains in the Netherlands, this may also be a problem that needs more attention in other countries. For example, countries wherein GPs are certified to give advice on sick leave also experience difficulties to assess the functioning of an individual in all life domains [32]. Therefore, recommendations in this study to address problems on multiple life domains may also apply to other countries. This study also showed that it is very hard to convince employers to invest in prevention. If we want employers to invest more in prevention, more knowledge and awareness must be created on the potential benefits of prevention with a focus on the return of investment for employers. Financial incentives or other forms of support may also be helpful, wherein the role of trade organizations, independent of employers, in facilitating preventive services should also be explored. However, curative healthcare also needs to invest more in prevention, as they, similar to occupational healthcare, invest too little in prevention. Thus, a societal change with more attention for and investments in prevention is required to address problems on multiple life domains at an early stage.

## Conclusions

This study provides valuable information on contextual factors that are important for implementation of preventive interventions that consider multiple life domains among employees with a lower SEP. The results also show the challenges of implementing these type of interventions in occupational health practice. Employees with a lower SEP and organizations employing them are difficult to reach for preventive health interventions. It is a challenge to convince stakeholders of the added value to preventively address and solve problems on multiple life domains. Moreover, the responsibilities for solving problems on multiple life domains are unclear. Many different stakeholders in organizations (e.g. supervisors), occupational healthcare (e.g. OPs), but also in curative healthcare (e.g. GPs) need to be involved and collaborate to effectively address problems on multiple life domains. Due to the complex systems in place, measures that lay beyond interventions should be taken into account to ensure the feasibility of these type of interventions in practice. It may even require adjustments to existing policies and procedures in occupational health practice.

## Supplementary Information

Below is the link to the electronic supplementary material.

Supplementary material 1 (DOCX 20.3 kb)

## Data Availability

The data generated and analyzed during the current study are not publicly available. The data consist of transcripts of interviews which contain identifying information, and are therefore sensitive to privacy issues. The data are available from the corresponding author on reasonable request.
