# Correction to: A Context Analysis with Stakeholders’ Views for Future Implementation of Interventions to Prevent Health Problems Among Employees with a Lower Socioeconomic Position

**DOI:** 10.1007/s10926-022-10040-z

**Published:** 2022-04-08

**Authors:** R. Schaap, F. G. Schaafsma, M. A. Huysmans, A. R. Bosma, C. R. L. Boot, J. R. Anema

**Affiliations:** Department of Public and Occupational Health, Amsterdam Public Health Research Institute, Amsterdam UMC, Vrije Universiteit Amsterdam, Van der Boechorststraat 7, 1081 BT Amsterdam, The Netherlands

## Correction to: Journal of Occupational Rehabilitation 10.1007/s10926-021-10010-x

In this article one of the following author last name Huijsmans, M.A. contains a typo.

For Huijsmans, M.A. it should not be ‘ij’, but an ‘y’.

The correct name is Huysmans, M.A.

The original article has been updated.

