# Supplementary material for: A Context Analysis with Stakeholders’ Views for Future Implementation of Interventions to Prevent Health Problems Among Employees with a Lower Socioeconomic Position
Source: J Occup Rehabil. 2021 Nov 3;32(3):438–51. doi: 10.1007/s10926-021-10010-x (PMC8564794; doi:10.1007/s10926-021-10010-x)
Supplement: Supplementary file 1 — Supplementary material 1 (DOCX 20.3 kb) [file 10926_2021_10010_MOESM1_ESM.docx]

**Supplementary material**

*Title*: A context analysis with stakeholders’ views for future implementation of interventions to prevent health problems among employees with a lower socioeconomic position

*Journal:* Journal of Occupational Rehabilitation

*Authors*: R. Schaap, F.G. Schaafsma, M.A. Huysmans, A.R. Bosma, C.R.L. Boot, J.R. Anema

*Affiliation:* Amsterdam UMC, Vrije Universiteit Amsterdam, Department of Public and Occupational Health, Amsterdam Public Health research institute, Amsterdam, Van der Boechorststraat 7, 1081 BT Amsterdam, The Netherlands.

*Corresponding author:* Rosanne Schaap, r.schaap@amsterdamumc.nl

**Table 1. Overview of themes, sub-themes and codes**

| **Theme** | **Sub-themes** | **Codes** | **Stakeholder** |
| --- | --- | --- | --- |
| The importance of addressing problems on multiple life domains among employees with a lower SEP | Number of problems higher among employees with a lower SEP | More often an accumulation of problems | Recognized as a problem among employees with a lower SEP: organization 1x, occupational health service 3x, socio-political macro level 5x |
|  |  | More often problems outside the workplace |  |
|  |  | More often unhealthy lifestyles |  |
|  |  | More often financial problems |  |
|  |  | Problems on multiple life domains more often among employees with a lower SEP |  |
|  | Reasons to focus more on employees with a lower SEP | Addressing problems on multiple life domains of importance for all employees, but employees with a lower SEP require more attention/support   - Problems on multiple life domains also among employees with a high SEP - Employees with a lower SEP have less problem solving skills | Important problem to address among employees with a lower SEP: organization 1x, occupational health service 2x, socio-political macro level 4x |
|  |  | Problems on multiple life domains difficult to solve (in time) among employees with a lower SEP |  |
|  | Characteristics employees with a lower SEP associated with problems on multiple life domains | Difficulties with finding help/finding solutions | Difficult problem to address among employees with a lower SEP: organization 3x, occupational health service 2x, socio-political macro level 7x |
|  |  | Lack of support/help from environment |  |
|  |  | Difficult to identify problems/less ability to reflect on problems |  |
|  |  | Difficult to motivate for interventions/do no ask for help   - Do not want to talk about problems - Employees eventually decide for themselves if they want to use the help or support |  |
| **Theme** | **Sub-themes** | **Codes en sub-codes** | **Stakeholder** |
| Unclarity of responsibilities for solving problems on multiple life domains | Occupational health services depend their services on the demands of employers | Occupational health service not feeling responsible   - Occupational health service commercial organization that sells services to employers - Services for occupational health eventually determined by the employer - Occupational health service advisor for employer | No responsibility to solve problems on multiple life domains: occupational health service 3x |
|  | Employers’ responsibility influences the manner in which problems outside the workplace are addressed | Employer not feeling responsible   - Employers place the cause for problems outside the workplace - SME employers are not feeling responsible - SME employers no expertise on occupational health - SME employer occupational healthcare arranged with occupational health service | Employers not responsible: occupational health service 2x, socio-political macro level 3x |
|  |  | Employer feeling responsible   - Problems outside the workplace are solved by external services/interventions - Employer facilitate solutions for problems outside the workplace | Employer responsible: organization 3x, occupational health service 2x, socio-political macro level 3x |
|  | Factors that influence the responsibility of employers | Depending on employers seeing their employees as valuable | Occupational health service 1x, socio-political macro level 2x |
|  |  | Depending on the financial resources that are available | Occupational health service 2x, socio-political macro level 1x |
|  | No shared responsibility between employers and employees | Employee is eventually responsible to solve problems   - Employer/professionals offer tools, do not solve problems | Shared responsibility: organization 1x, socio-political macro level 2x |
|  |  | Influence of employees on occupational health policies dependent on Works Council/type of organization   - Employees with a lower SEP smaller amount of influence on occupational health policies | No influence of employees: socio-political macro level 2x |
|  | No responsibility experienced in occupational and curative healthcare | General practitioner and occupational health physician both not responsible to solve all problems on multiple life domains | No responsibility in occupational and curative healthcare: socio-political macro level 4x |
|  |  | Occupational medicine must focus on problems at the workplace |  |
|  |  | General medicine must focus on health complaints |  |
|  |  | General practitioner have limited expertise with problems at the workplace |  |
|  |  | General practitioners have not enough time for problems at the workplace   - General practice nurses have more time and can collaborate with occupational health professionals |  |
|  | No responsibility for trade organizations | Trade organizations have an advisory role/share knowledge, do not determine which services are provided | All trade organizations; KOM, Volandis, OVAL |
| **Theme** | **Sub-themes** | **Codes en sub-codes** | **Stakeholder** |
| Necessity of better collaboration between occupational and curative health care | Two pathways for identifying problems on multiple life domains | Problems on multiple life domains can be discussed at the workplace   - Depending on organizational culture | Organization 3x, occupational health service 4x, socio-political macro level 4x |
|  |  | Problems on multiple life domains can/should be discussed in curative health care | Occupational health service 2x, socio-political macro level 2x |
|  | Collaboration between occupational and curative health care is important | Collaboration between occupational and curative healthcare is important   - General practitioner/general practice nurse first person to contact in case of health complaints - Not everyone has access to occupational healthcare | Collaboration is important: occupational health service 2x, socio-political macro level 6x |
|  | Collaboration is not facilitated | Collaboration professionals occupational and curative healthcare is difficult   - Occupational medicine separate from curative health care is a barrier for collaboration - Privacy regulation is a barrier for exchanging information between professionals/organizations | Collaboration is experienced as difficult: occupational health service 2x, socio-political macro level 5x |
|  | Improve collaboration between occupational and curative healthcare by integral care | Organize healthcare around an individual employee (network care) | Improve collaboration by integral care: occupational health service 2x, socio-political macro level 5x |
|  |  | Occupational physician in curative healthcare |  |
|  |  | Take into account work factors in curative healthcare |  |
| **Theme** | **Sub-themes** | **Codes en sub-codes** | **Stakeholder** |
| Insufficient investments in prevention by employers | More attention needed for prevention | Prevention is an important priority   - Much attention for prevention | Prevention is important: Organization 3x, occupational health service 4x, socio-political macro level 5x |
|  |  | More attention needed for prevention   - More attention needed for prevention in education of health professionals - Trade organizations can give attention to prevention among employers | More attention needed: socio-political macro level 4x |
|  | Insufficient attention for prevention in contracts | Prevention at the workplace dependent of the contract between employer and occupational health service   - Preventive services often not included in the basic contracts - Contracts focus on the guidance of employees on sick leave - Attention employer shifted to sick leave due to the gatekeeper act - Occupational health professionals not always involved in forming of contracts - Occupational health professionals more involved in the formation of contracts, more likely to be used for preventive services | Prevention insufficient in contracts: organization 1x, occupational health service 3x, socio-political macro level 4x |
|  | Insufficient investments in prevention | Employers are less/not willing to spend money on prevention | Occupational health service 1x, socio-political macro level 4x |
|  |  | Employers are more willing to spend money on prevention | Occupational health service 2x |
|  | Reasons for insufficient investments in prevention | Prevention dependent on the financial resources that are available   - Smaller organizations and/or organizations in crisis have less (financial) resources | Availability of money: organization 2x, occupational health service 3x, socio-political macro level 5x |
|  |  | Prevention dependent on employers seeing their employees as valuable | Socio-political macro level 4x |
|  |  | Prevention dependent on the visibility of results   - Results of prevention are unclear - Results of prevention not quantifiable - Employers focus on short term results | Visibility of results: organization 1x, occupational health service 2x, socio-political macro level 6x |
|  |  | Employer is not/less aware of the benefits of prevention | Not aware of benefits: organization 1x, occupational health service 1x, socio-political macro level 3x |
|  |  | Employer is aware of the benefits of prevention   - We only act if there is a problem | Aware of benefits: organization 2x, occupational health service 1x, socio-political macro level 2x |
|  |  | Prevention dependent on support of key stakeholders in the organization | Organization 2x, Occupational health service, socio-political macro level 4x |
|  | Increase investments in prevention | Improve prevention by working out business cases and with innovation in preventive services | Socio-political macro level 4x |
| **Theme** | **Sub-themes** | **Codes en sub-codes** | **Stakeholder** |
| Difficulties in early identification of employees at risk for health problems | Methods for identification focus on indicated prevention | Identification of employees at risk based on monitoring, sick leave, stop sign model, social medical team consultation (SMT), preventive medical examination (PMO)   - Employees are identified when they are experiencing problems | Focus on indicated prevention: Organization 3x, occupational health service 4x, socio-political macro level 1x |
|  | Occupational health physicians mainly used for guidance of employees on sick leave | Perform regularly conversations with employees to preventively address problems on multiple life domains   - Occupational physician limited available to solve problems on multiple life domains preventively - Occupational social workers/occupational nurses preventively more available to solve problems on multiple life domains | Occupational health physicians mainly used for guidance of employees on sick leave: organization 1x, occupational health service 1x, socio-political macro level 3x |
|  | Individual preventive conversations with occupational health professionals and follow-up interventions | Individual preventive conversations/follow-up interventions with occupational health professionals are (more often) performed | Organization 2x, occupational health service 4x, socio-political macro level 2x |
|  |  | Individual preventive conversations/follow-up interventions are not often performed   - Dependent on the extent to which an organization wants to invest | Occupational health service 2x, socio-political macro level 3x |
| **Theme** | **Sub-themes** | **Codes en sub-codes** | **Stakeholder** |
| Risk of conflicting role for supervisors in addressing problems on multiple life domains | Supervisors play an important role in identifying and discussing problems preventively | Supervisors play an important role in the early identification of employees at risk for health problems   - Supervisors play an important role in referring employees to an occupational health professionals on time | Supervisors play an important role: organization 2x, occupational health service 3x, socio-political macro level 1x |
|  |  | Supervisors have preventive conversations with employees |  |
|  |  | Much attention in organizations and occupational health services in training of supervisors in early identification of problems and performing preventive conversations with employees. |  |
|  |  | Self-management model: improve responsibility of supervisors on guidance of employees health and safety |  |
|  | Reasons for not using supervisors in identifying and discussing problems preventively | Self-management model; employees disadvantaged   - Risk that supervisors take on the role of the occupational health professional - Risk that supervisors take advantage of privacy-sensitive information of employees - Risk that to come in contact with occupational health professionals is more difficult or too late - Occupational health professional important to advice employees independently of other interests | Not using supervisors: organization 3x, occupational health service 3x, socio-political macro level 3x |
|  |  | Self-management model difficult, as supervisors are not allowed to ask employees everything   - Usually employees discuss everything with their supervisor - Privacy regulations to discuss problems on multiple life domains unclear |  |
